# Neuromuscular control: from a biomechanist's perspective

**DOI:** 10.3389/fspor.2023.1217009

**Published:** 2023-07-05

**Authors:** Daanish M. Mulla, Peter J. Keir

**Affiliations:** Department of Kinesiology, Occupational Biomechanics Laboratory, McMaster University, Hamilton, ON, Canada

**Keywords:** neuromechanics, optimization, synergy, musculoskeletal modelling, simulation, muscle, sensorimotor

## Abstract

Understanding neural control of movement necessitates a collaborative approach between many disciplines, including biomechanics, neuroscience, and motor control. Biomechanics grounds us to the laws of physics that our musculoskeletal system must obey. Neuroscience reveals the inner workings of our nervous system that functions to control our body. Motor control investigates the coordinated motor behaviours we display when interacting with our environment. The combined efforts across the many disciplines aimed at understanding human movement has resulted in a rich and rapidly growing body of literature overflowing with theories, models, and experimental paradigms. As a result, gathering knowledge and drawing connections between the overlapping but seemingly disparate fields can be an overwhelming endeavour. This review paper evolved as a need for us to learn of the diverse perspectives underlying current understanding of neuromuscular control. The purpose of our review paper is to integrate ideas from biomechanics, neuroscience, and motor control to better understand how we voluntarily control our muscles. As biomechanists, we approach this paper starting from a biomechanical modelling framework. We first define the theoretical solutions (i.e., muscle activity patterns) that an individual could feasibly use to complete a motor task. The theoretical solutions will be compared to experimental findings and reveal that individuals display structured muscle activity patterns that do not span the entire theoretical solution space. Prevalent neuromuscular control theories will be discussed in length, highlighting optimality, probabilistic principles, and neuromechanical constraints, that may guide individuals to families of muscle activity solutions within what is theoretically possible. Our intention is for this paper to serve as a primer for the neuromuscular control scientific community by introducing and integrating many of the ideas common across disciplines today, as well as inspire future work to improve the representation of neural control in biomechanical models.

## 1. Introduction

A fundamental challenge facing biomechanists and neuroscientists alike is understanding neuromuscular control. For a given task, an individual can have several movement strategies available for facilitating performance (1). Each movement has a potentially infinite combination of feasible force solutions across muscles (2, 3). A single muscle can be composed of multiple anatomical regions (4–6), each with their own mechanical functions that are innervated by hundreds of sensory and motor neurons (7). How the neuromusculoskeletal system navigates this complex, abundant landscape of possibilities at several levels while producing smooth, voluntary movement with ease is remarkable. The complexity of sensorimotor control can be especially appreciated when considering the tremendous challenges that scientists and engineers encounter while attempting to design biologically inspired robots and machines (8–10). The upper limb is a particularly intricate system as it encompasses more than 70 muscles and 34 rotational degrees of freedom, enabling a wide range of movement and manual dexterity unparalleled by any other part of the human body (11, 12). A thorough understanding of neuromuscular control of the arm will aid in the design of robotic and prosthetic control, and perhaps more importantly, inform strategies for improving task performance, skill acquisition, and rehabilitative treatments for movement disorders.

**Figure 1:**
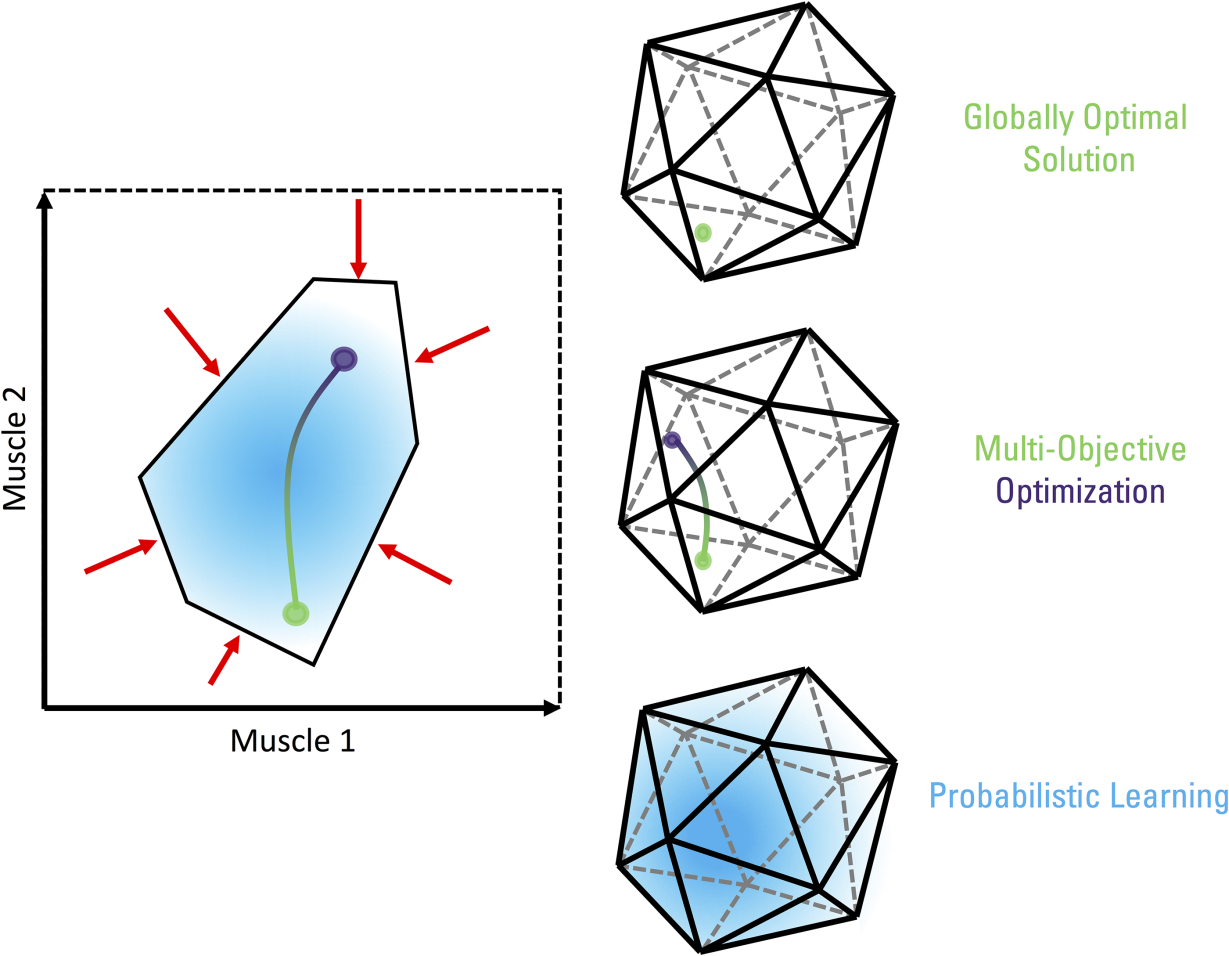
Conceptual visualization of a feasible solution space of muscle activations for a task at a single instant in time. The example on the left displays the activation of two muscles as part of a hypothesized limb with *n* muscles (*n* > 2) and m kinematic degrees of freedom, where *n* > m, as representative of human limbs. Unconstrained, the activation level for both these muscles can range from 0 (no activation) to 1 (maximum activation). Neural and mechanical factors constrain (red arrows) the activation patterns within a subset of these ranges (black polytope), as required to complete the task. The set of all muscle activation patterns within this polytope represent the feasible solution space. The feasible solution space will be a convex polytope in the case of linear constraints that do not produce a null set. Nonlinear constraints could produce nonconvex feasible solution spaces with curved boundaries. Without additional considerations, the neuromusculoskeletal system is free to vary muscle activations within this space as all feasible solutions are treated equally while keeping task performance constant. Certain solutions, however, may be preferred as guided by hypothesized sensorimotor control principles. Optimization aims to identify the single point within the feasible space that best attain the task performance under such principles. For example, most biomechanical models assume effort minimization, thus, solving for the muscle activation pattern that affords the lowest effort (green circle). In contrast, a system requiring maximum stiffness would necessitate greater muscle activations (and forces) as a means of minimizing displacements to perturbations, leading to a drastically different solution (purple circle). If both effort and stiffness are optimized (i.e., multi-objective optimization), a landscape of optimal solutions is formed depending on the relative weighting between effort and stiffness (green-purple line) as there is rarely a single feasible solution that globally optimizes multiple objectives. Note that we are assuming non-linear cost functions (as are typical in the biomechanics and motor control literature), where optimal solutions may lie inside or on the boundary of the feasible solution space. In the case of linear cost functions, optimal solutions (if they exist) will lie only on the boundary of the solution space. “Good-enough” solutions are suboptimal and are found in the neighbourhood of an optimal solution in the cost function landscape. In this case, a “good enough” solution would generate a combination of effort and stiffness costs that would be comparable to the globally optimal solution but is not the best combination that could be found. A probabilistic perspective to neuromuscular control is driven by exploring the feasible solution space with trial-and-error, such as Markov chains, simulated annealing, and genetic algorithms. If shaped by optimality principles and provided with sufficient exploration, the probability density of muscle activation patterns will converge towards optimal solutions with motor learning (blue gradient cloud). The examples on the right are visual depictions of the same neuromuscular control principles for a higher dimension feasible solution space.

Several theories and conceptual frameworks have been proposed to explain and understand neuromuscular control. Recently, Valero-Cuevas (13) and then Cohn et al. (2) presented “Feasibility Theory” as an approach grounded in biomechanical modelling that encompasses prior neuromuscular control theories (Figure 1). Feasibility Theory aims to understand neuromuscular control by defining the set of all theoretically feasible solutions for a task, exploring the structure of the solution space, and how individuals inhabit and navigate the space. When faced with a task, an individual's neuromusculoskeletal system is met with several constraints. These constraints include neural (e.g., hard-wire pathways) and mechanical factors (e.g., task demands, musculoskeletal limb properties) (14–18). Together, the constraints define the feasible solution space of muscle activations (i.e., red arrows and black polytope in Figure 1). An ongoing debate is whether muscle synergies, described as a functional unit of muscle groups with weighted co-activation, are an indirect consequence of constraints and optimality principles shaping the relationships across muscles (i.e., descriptive synergies) or are a specific constraint by the nervous system aiming to reduce control into a lower dimensional problem (i.e., prescriptive synergies) (13, 19–22). Nevertheless, the neuromusculoskeletal system can theoretically traverse the feasible solution space without any immediate impact on task performance. In other words, accounting for the neuromechanical properties of the limb and task demands, there are several ways to activate muscles with no difference in net joint torques, resulting in identical endpoint wrench (forces and torques) of the limb (13). The solution space is comparable to the uncontrolled manifold hypothesis, where the motor system can take advantage of the abundance of solutions by allowing elements (in this case, muscle activations) to freely vary within this subspace while keeping a select performance variable(s), such as endpoint forces and torques, constant (23, 24).

It is hypothesized that all feasible solutions are not equivalent and certain solutions may be preferentially selected by overarching optimality principles. Traditionally in biomechanics and motor control, this is addressed as the degrees of freedom, redundancy, abundancy, underdetermined, indeterminacy, or load-sharing problem. It is commonly resolved using optimization (e.g., static optimization, optimal control) to determine a single solution within the feasible space that best meets a criterion of choice. Note that optimization methods using gradient-based search for minima (e.g., static optimization, optimal control) are a special case of machine learning. As described in detail by Valero-Cuevas et al. (25), computational methods that allow such explorations of feasible spaces are a combination of machine learning, control theory, and estimation detection theory. This combination has resulted in methodologies including reinforcement learning, unsupervised learning, optimal control, and many others being developed. However, as traditionally applied to neuromuscular control to find unique solutions, the choice of objective function to optimize varies but is often related to minimizing “effort” (i.e., green circle in Figure 1) (26–30). Others propose the use of multiple objective functions (31, 32), such as the additional consideration for limb stiffness (i.e., purple circle in Figure 1) (33, 34). In a multi-objective optimization, there is rarely a single global solution that will simultaneously optimize multiple objectives. This leads to a landscape of optimal solutions depending on the relative weights given to each objective (i.e., green-purple line in Figure 1). Optimal feedback control theory is a specific application of closed-loop optimal control, where a control policy (such as a gain matrix) is sought to determine the control signals (i.e., motor commands) that maximize task goals in addition to minimize effort (amongst other possible variables) while integrating state-dependent sensory (feedback) information with an internal feedforward model (35–38). What remains relatively less studied is evaluating the landscape of suboptimal solutions in the neighbourhood of the globally optimal solution (34, 39). Through exploration of the feasible solution space via trial-and-error, a probabilistic representation of motor patterns can be formed (40, 41). This probabilistic representation characterizes the degree of belief (or uncertainty) in which motor solutions may work for completing a given task. Such probabilistic representations are based on prior experiences (habitual behaviour) and can be further shaped by optimality principles. A probabilistic trial-and-error search guided by a fitness criterion functions as a Markov chain (42). Repeatedly exploring the solution space can lead to finding more rewarding solutions (e.g., less effortful solutions for accomplishing the same task) that are reinforced and exploited over time (i.e., blue gradient cloud of solutions in Figure 1). With motor learning, individuals may then converge upon *the* optimal solution, or alternatively, one of several *approximately* optimal solutions [or “good-enough” as coined in (39)] for a task at hand.

By identifying the set of all feasible motor solutions for a task, Feasibility Theory provides an elegant approach that embraces the complementary and competing perspectives of several neuromuscular control theories (e.g., muscle synergies, uncontrolled manifold hypothesis, optimization, probabilistic control). In the process, it naturally raises several fascinating questions:

• What are the specific neural and mechanical constraints that define the feasible solution space?
• Given these constraints, how do measured muscle activity patterns compare to feasible solution space of muscle activations? Do measured muscle activity patterns occupy a narrow or wide region within what is theoretically feasible?
• What hypothesized objective(s) does the neuromusculoskeletal system aim to optimize?
• Can different weightings across multiple objectives help explain within- and between-subject variability in motor patterns?
• With learning, do muscle activity patterns better approximate optimal solutions?
• Can variations in arriving at different “good enough” solutions help explain within- and between-subject variability in motor patterns?

Although the questions posed above are recognized and tackled in the literature, finding answers (especially for individuals starting out in this field) can be challenging because insights are often provided across several papers crossing many disciplines. As such, the purpose of this review is to explore many of these questions by integrating ideas from biomechanics, neuroscience, and motor control to understand how we voluntarily control our muscles. In particular, we aim to provide a broad presentation of the challenges and opportunities in this field, incorporating both modelling and experimental evidence, to allow others to make sense of the current debates and pitfalls, and help the community make progress in the future. We begin the paper by first formulating the degrees of problem using biomechanical modelling (Section 2). Motivated by the recently proposed Feasibility Theory (2) and the modelling foundations laid out in the textbook “Fundamentals of Neuromechanics” (13), we will use the concept of feasible solution spaces to develop an intuition and understanding of the degrees of freedom problem, as well as discuss earlier uses of solution space approaches in biomechanics and motor control (Section 3 and 4). The feasible solution space defines the set of all theoretical solutions that an individual's motor system has available to perform a task. The focus throughout this paper will be on the degrees of freedom problem at the muscle level, especially during static tasks, with particular emphasis on the upper extremity. The set of all theoretical possible solutions based on the feasible solution space will be compared with individuals' actual motor behaviour based on experimental data (Section 5). It will be highlighted that individuals exhibit structured patterns in their motor behaviour which inhabit only small volumes of the feasible solution space, raising the possibility of overarching neuromuscular control principles and/or neuromechanical constraints that may be governing motor behaviour. We will discuss dominant neuromuscular control theories, highlighting optimality, probabilistic principles, and neuromechanical constraints that are used to identify families of solutions in the feasible solution space (Section 6). The incompatibilities between current optimization-based model predictions and experimental data will be discussed and used as an impetus for future studies to account for additional neuromechanical factors and principles that may be shaping muscle activity patterns but are unaccounted for in current biomechanical models.

## 2. Biomechanical modelling and the degrees of freedom problem

The equations of motion for a rigid body with *m* number of kinematic degrees of freedom can be expressed in linear algebra form as:

(1) $$\begin{array}{c} \tau =M(q)\ddot{q}+C(q,\dot{q})+G(q)+J^{T}\tau_{\mathrm{end}}\; \end{array}$$

where τ is the *m* × 1 vector of the generalized joint forces (i.e., net joint forces and torques); *q*,$\;\dot{q},$ and $\ddot{q}$ are the generalized joint positions, velocities, and accelerations, respectively; M(q) is the *m* × *m* inertia matrix; $C(q,\dot{q})$ is the *m* × 1 vector of Coriolis forces that includes the velocity-related terms; G(q) is the *m* × 1 vector of gravitational forces; $\tau_{\mathrm{end}}$ is the vector of forces and torques at the endpoint of the limb; and $J^{T}$ is the Jacobian matrix mapping endpoint to joint-level forces and torques.

The net joint forces and torques are balanced by the internal force carrying structures at the joints, which mainly include the musculotendon units, ligaments, and joint contact forces. As musculotendon units are the main contributors to net joint forces and torques, especially for postures in the mid range of motion typical of many tasks, most biomechanical models only include muscles. For a rigid body system with *n* number of muscles, the balance of net joint torques by musculotendon units can be expressed as:

(2) $$\begin{array}{c} \tau =\;R(q)F_{0}^{MT}a \end{array}$$

where R(q) is the *m* × *n* matrix of muscle moment arms; $F_{0}^{MT}$ is the *n* × *n* diagonal matrix of maximum musculotendon forces; and *a* is the *n* × 1 vector of muscle activations which is commonly assumed to range from 0 (no activation) to 1 (maximum activation). For the purposes of this paper, the term muscle activation will be used interchangeably to represent the theoretical neural commands sent to muscles and measurements made using electromyography (EMG), although caution is advised when interpreting neural commands through EMG (43, 44). Combining Equations 2, 3 allows us to map a transformation between muscle activations and the endpoint vector of forces and torques:

(3) $$\begin{array}{c} R(q)F^{MT}a=M(q)\ddot{q}+C(q,\dot{q})+G(q)+J^{T}\tau_{\mathrm{end}} \end{array}$$

Human limbs have a greater number of muscles than kinematic degrees of freedom (*n* > *m*), which in turn are typically greater than the number of elements in the endpoint force and torque vector. As a result, Equations 2, 3 express an important concept that has challenged the area of biomechanical modelling for the past few decades and will be central to this paper. These equations represent a transformation from a higher dimensional space (muscle activations) to a lower dimensional space (net joint torques and endpoint force vector). In other words, there are more unknowns than knowns. Mathematically, Equations 2, 3 represent an underdetermined problem, allowing for several non-unique solutions (i.e., combinations of muscle activations), if they exist, for most tasks. In biomechanics and motor control, this is famously termed the degrees of freedom problem [often traced originally to the work by Nikolai Bernstein (1)] and is also otherwise known as the muscle/motor redundancy (or abundancy), statically indeterminate, or load-sharing problem.

Before proceeding with the biomechanical modelling formulation presented above, a few remarks are required to set the context for the rest of the paper. Foremost, the biomechanical problem noted across Equations 1–3 traditionally focuses on determining muscle activity patterns to balance the net joint torques. However, human joints do not only exhibit rotational but also translational degrees of freedom. Furthermore, torques are not the only mechanical variable guiding muscle coordination. Motor behaviour can be driven by other mechanical factors. In particular, stability considerations may be essential from a task performance and injury prevention perspective (45, 46). In fact, the neuromechanical control of motion vs. force are distinct and can be incompatible with each other, with impedance control postulated by some researchers as an approach to bridge between the two (47–50). The flip side of the control of joint torques being underdetermined is that the control of movement driven by afferented muscles is overdetermined (13, 51). Specifically, the change in angles of a few joints determines the change in lengths of many musculotendon units. Finally, biomechanical models commonly assume musculotendon units to act mechanically and neurophysiologically independent of each other, with the prescribed activation and consequent forces generated in one muscle unaffected by other muscles. Our intention to make these caveats clear from the outset is to caution readers to keep these modelling assumptions and viewpoints in the back of their mind. For now, we begin this paper by adopting the traditional modelling framework (e.g., focusing on balancing joint torques, assuming independent control of musculotendon units) because it is the dominant approach in the field of biomechanics and allows us to be on the same starting page with the majority of biomechanical models and modelling programs currently available. Starting broadly also allows researchers to develop more detailed models as they see fit to answer their own neuromuscular control research questions of interest. Over the course of this paper, we will unravel many of the neuromechanical factors that are not always incorporated in traditional biomechanical models but may be essential for shaping muscle activity patterns.

## 3. Theoretically feasible solutions

The degrees of freedom problem fundamentally inquires about how the nervous system navigates and selects solutions from the set of all possible combinations of muscle activations (i.e., feasible solution space) that can theoretically be used to perform a task. For about half a century, since the 1970s, the standard approach in biomechanics has been to use optimization to solve for the single solution within the feasible solution space that best matches a hypothesized control principle approximating how the nervous system coordinates muscles (16, 18, 27, 30, 52–55). Before taking a closer look at the use of optimization, the feasible solution space of muscle activations itself warrants a detailed discussion as it lies at the heart of the degrees of freedom problem. Doing so will allow us to place experimental data of how humans behave within the context of what is theoretically possible based on biomechanical models. Similar approaches (uncontrolled manifold, goal-equivalent manifold, and tolerance-noise-covariation) have been undertaken in the field of motor control, where theoretical solution spaces of task execution variables (e.g., angle and velocity at release point during throwing) are compared to individual performance to reveal factors associated with motor learning (23, 24, 56–59). The feasible solution space can be used to generate null models to statistically test whether observed behaviour exhibit certain features that would otherwise be unexpected if individuals randomly navigated (i.e., sampled) the entire space (60). In the process, it enables researchers to evaluate competing hypotheses on which neuromechanical factors (e.g., constraints, optimality principles, probabilistic learning) are shaping the emergence of the specific muscle activity patterns from the broader landscape of all possible solutions, and how this may vary across different contexts, such as with learning, fatigue, neuromuscular impairments, or following surgical interventions (2, 16). In general, there are two approaches used to identify the feasible solution space of muscle activations: analytical and numerical. Both approaches have elegant graphical interpretations that further our understanding of the degrees of freedom problem (16, 61).

**Figure 2:**
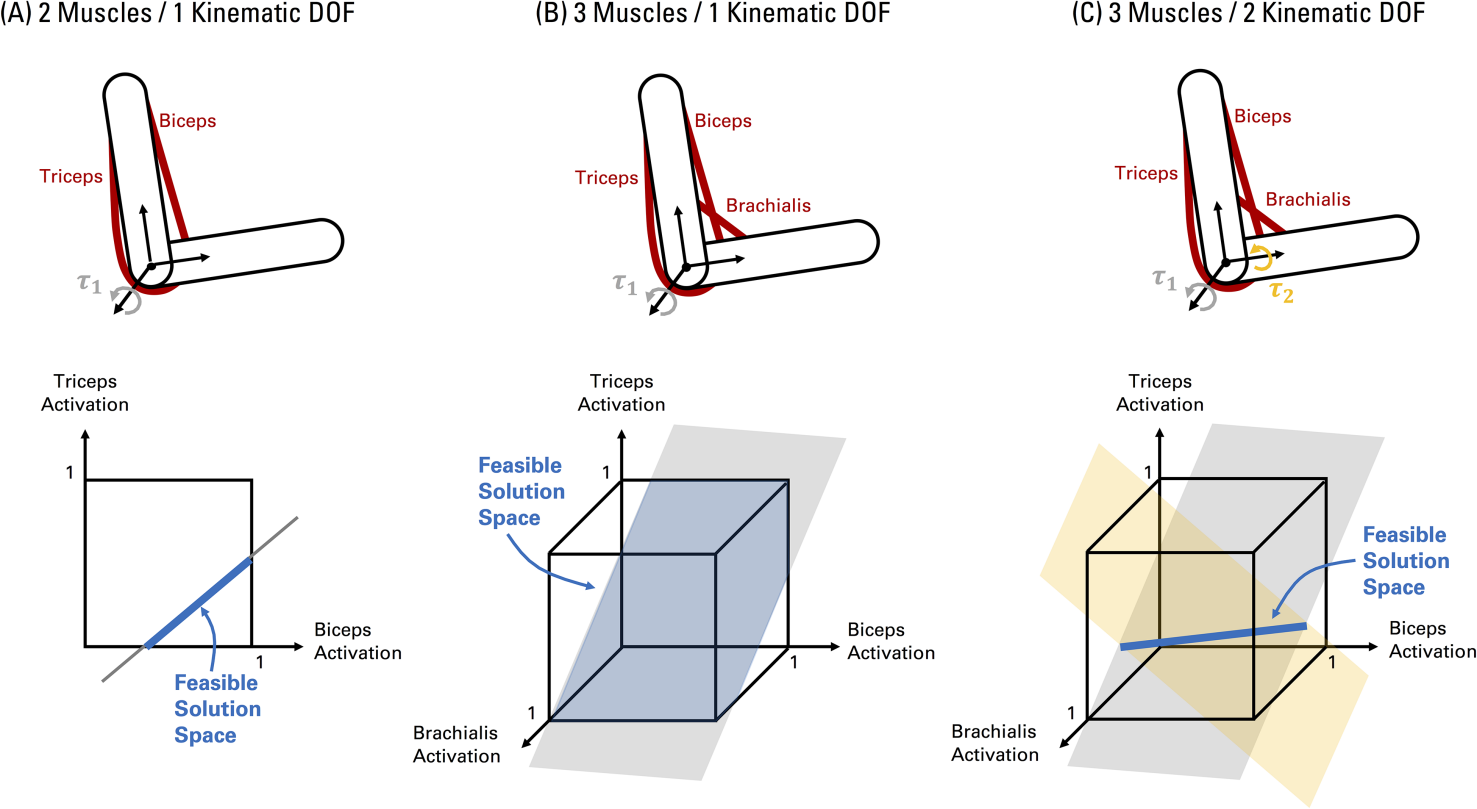
Visualizing the feasible solution space of muscle activations for simple elbow musculoskeletal models. (**A**) A system with 2 muscles and 1 kinematic degree of freedom. Given two muscles, the muscle activation space is two-dimensional as shown in the graph. The black border in the graph represents the bounds on muscle activations from 0 (no activation) to 1 (full activation) (Equation 5). The grey line represents the combination of muscle activations that can meet the net joint torque ($\tau_{1}$) requirement at the single kinematic degree of freedom (Equation 4). Note that the intercept and slope of the line is defined by the net joint torque, muscle moment arms, and maximum musculotendon forces. The feasible solution space of muscle activations is the segment of the line (shaded in blue) inside the muscle activation bounds. (**B**) The feasible solution space of a system with 3 muscles and 1 kinematic degree of freedom can be represented by the portion of a plane (Equation 6) within the boundaries of a 3D unit cube (Equation 7), as shaded by the blue area. (**C**) The feasible solution space of a system with 3 muscles and 2 kinematic degrees of freedom can be represented by the intersection of two planes, creating a line (shaded in blue) in 3D space. The shape and orientation of the planes in (**B, C**) are defined by the net joint torques $\left(\tau_{1},\tau_{2}\right)$, muscle moment arms, and maximum musculotendon forces.

Analytical approaches characterize the exact feasible solution space. For example, from Equation 2, we can mathematically describe a system with 2 muscles and 1 kinematic degree of freedom as:

(4) $$\begin{array}{c} \tau_{1}=\;(r_{1}f_{0}^{MT_{1}})a_{1}+(r_{2}f_{0}^{MT_{2}})a_{2}\; \end{array}$$

(5) $$\begin{array}{c} 0\;\leq a_{1},a_{2}\leq 1\; \end{array}$$

where $r_{n}$, $f_{0}^{MT_{n}}$, and $a_{n}$, refer to the moment arm, maximum musculotendon force, and activation of each muscle (*n*) respectively, which balance the net joint torque $\tau_{1}$. Equation 4 represents a linear mechanical constraint to meet task demands (i.e., net joint torque torque) based on the musculoskeletal properties of the limb (i.e., moment arms and maximum musculotendon forces). Equation 5 represents the inequality constraints bounding muscle activations from 0 (no activation) to 1 (maximum activation). Thus, in this case, the feasible solution space of muscle activations is a line defined by the mechanical constraint (Equation 4) within a unit square representing the muscle activation boundaries (Figure 2A). Increasing the count to 3 muscles give us:

(6) $$\begin{array}{c} \tau_{1}=\;(r_{1}f_{0}^{MT_{1}})a_{1}+(r_{2}f_{0}^{MT_{2}})a_{2}+(r_{3}f_{0}^{MT_{3}})a_{3} \end{array}$$

(7) $$\begin{array}{c} 0\;\leq a_{1},a_{2},a_{3}\leq 1\; \end{array}$$

In this case, the feasible solution space of muscle activations is visualized as a plane (Equation 6) within the boundaries of a unit cube (Equation 7) in 3D space (Figure 2B), as originally depicted by Crowninshield & Brand (26, 27) for the case of 3 muscles for an isometric elbow flexion task. If the number of kinematic degrees of freedom increase to 2 (e.g., elbow flexion-extension and forearm pronation-supination), the feasible solution space of muscle activations is then the intersection (if it exists) of two planes in 3D space, creating a line if the planes are not co-planar (62) (Figure 2C). Interestingly, Crowninshield & Brand (26, 27) illustrated changes in the selection of optimal solutions within the feasible solution space of muscle activations based on the nature of the cost function, particularly shifting towards the interior regions promoting higher muscle co-activation when using non-linear cost functions motivated by physiological bases. In this vein, analytical approaches are effectively applied to identify the force-sharing relationships across muscles and contexts when muscle co-activation can or cannot be predicted depending on the task conditions and/or structure of the musculoskeletal model (e.g., number of joints and kinematic degrees of freedom, uniarticular vs. biarticular groups of agonists and antagonist muscles) (16, 63–69). However, these investigations are primarily proof of principle focused on simple models. Generalizing the analytical approach to increasing complex models is challenging to interpret and visualize, as the feasible solution space is the intersection of multiple hyperplanes (each representing a single kinematic degree of freedom) imbedded in a hypercube with the number of dimensions equal to the number of muscles, resulting in a high-dimensional convex polytope (in the case of linear constraints, see Figure 1 caption) (13). Computational geometry tools, such as vertex enumeration algorithms, can be used to determine the vertices of the resulting polytope (17), and thus, the range of possible activation levels for each muscle in more complex models. As a result, researchers may then identify muscles that are “necessary” or “redundant” based on if a given task can be completed without activating a given muscle (20, 62). Nevertheless, in the former study, the model was reduced in complexity (from 44 to 14 muscles) to yield a solvable problem (20). Thus, analytical methods quickly become intractable when modelling human limbs.

Numerical approaches find approximations to the feasible solution space that help overcome the difficulties with applying analytical methods to complex models. Numerical methods can be broadly grouped into two categories: optimization and stochastic. Optimization methods find muscle activity patterns within the feasible solution space that minimize and maximize the activation of one particular muscle [see Figure 2 from (3)], thus identifying the range of activations possible for that muscle. Iterating this optimization process across all muscles will yield the activation range for each muscle within the feasible solution space. This method has been applied to quasi-static feline hindlimb standing balance (3, 34, 70), human walking (71), and static pedalling endpoint forces (72). Note that optimization in the context of identifying the feasible solution space differs from the traditional use of optimization in biomechanics used to find a single solution to solve the load-sharing problem (see Section 6). Although optimization can be effectively applied to determine the boundaries of the feasible solution space, it does not reveal any details on the internal structure of the feasible solution space. In contrast, stochastic solutions randomly sample specific points within the feasible solution space (2, 73, 74). Stochastic methods may not identify the true boundaries of the muscle activation range, but if enough points within the space are uniformly sampled, it allows for a glimpse at the internal structure of the feasible solution space by identifying how muscles would need to activate in relation with each other under the biomechanical constraints of the task and limb [see Figure 3 from (2)]. Alternatively, the solution space can be explored deterministically by first identifying the vertices and sampling interspersed points between the vertices (75, 76). Importantly, numerical approaches can be applied to approximate the feasible solution space in highly complex models, ranging from 7 to 92 musculotendon actuators with 4–23 kinematic degrees of freedom across the studies cited above.

## 4. Structure of the feasible solution space

From our formulation of the feasible solution space, we can immediately recognize that the structure of the space (i.e., number of dimensions, size, boundaries, orientation) will be fundamentally shaped by the interaction between the musculotendon mechanics, musculoskeletal geometry, and task demands. Musculotendon mechanics defines the capacity of our muscles to generate and transmit forces to bones, and is determined by properties of the muscle and tendon such as the maximum isometric force, pennation angle, and force-length-velocity relationship. Musculoskeletal geometry is the spatial relationship between the musculotendon units and the skeletal structure (i.e., muscle lines of action and moment arms) that map musculotendon forces into joint and endpoint forces/torques. The term limb mechanics will be used to refer to the collective effects of musculotendon mechanics and musculoskeletal geometry. Task demands refer to the magnitude and direction of the endpoint forces/torques required by the limb to perform a given task. In our simple case of a 2 muscle, 1 degree of freedom system (Equation 5 and Figure 2A), the feasible solution space is defined as a line, with its y-intercept and slope dependent on the maximum musculotendon force (musculotendon mechanics), moment arms (musculoskeletal geometry), and net joint torque (task demands). Altogether, limb mechanics and task requirements represent biomechanical constraints that shape the feasible solution space, and hence the muscle activity patterns required to perform a task. As a consequence, muscle coordination is not an arbitrary recruitment of muscles that will produce stochastic relationships in muscle activity patterns. Instead, the feasible solution space will have intrinsic structure. Multivariate relationships and correlations between muscles will naturally emerge due to the biomechanical properties of the limb meeting the specific task demands at hand (2, 75).

Studies directly evaluating the structure of the muscle activation solution space observe a complex dependency on tasks and limbs. When performing sub-maximal tasks, the feasible solution space is generally wide, and can range to the natural limits for many muscles (i.e., activations can range from 0%–100%) (71); however, this space tends to decrease (i.e., smaller range of muscle activation levels) as task intensity increases (2, 3). The increased feasibility at sub-maximal levels is possible because muscles have more ways to co-contract to compensate and balance joint moments. As most daily tasks are sub-maximal, this suggests that there can be substantial opportunity for within-individual variation in muscle activity patterns despite no changes in posture. In addition to the task intensity, the precision required for a task, such as during fine motor activities, will influence the feasible solution space. Kutch & Valero-Cuevas (62) demonstrated that the range of feasible activations for index finger muscles were practically limitless when generating sub-maximal fingertip radial forces without considering off-axis fingertip forces. Enforcing hard constraints to the off-axis forces, as simulating a well-directed precision key pinch task, drastically limited the possible muscle activity patterns to a small subset of solutions. The implication is that muscle impairments resulting in decreased musculotendon force capacity will predominantly affect the ability to perform tasks requiring greater intensity and/or precision.

Robustness to perform tasks with muscle dysfunction may vary across limbs. Comparing across models of varying complexities, Sohn et al. (72) reported that the feasible endpoint forces that can be generated at the endpoint of a human leg is more robust to muscle impairments in models of decreasing kinematic complexity (i.e., lower number of kinematic degrees of freedom) and increased number of muscles. It was further suggested that a ratio of muscles to kinematic degrees of freedom can provide an estimate of the musculoskeletal redundancy across different limbs. In other words, systems with more kinematic degrees of freedom add constraints which decrease the number of possible muscle activity solutions for a given task. In contrast, more independently controllable musculotendon units increase the possible number of muscle activity solutions. As such, limbs with a *seemingly* higher degree of musculoskeletal abundancy (based on model choices, see next paragraph) may have greater flexibility in feasible muscle activity patterns. Hence, relationships emerging from the feasible solution space may be less structured compared to limbs with a smaller ratio of muscle to kinematic degrees of freedom, indicating a fundamental difference in neuromuscular control of limbs of varying complexities.

The findings from Sohn et al. (72) concurrently reveal the importance of model choices [for a detailed review, see (77)]. As an example, the shoulder can exhibit up to nine rotational degrees of freedom with additional translational degrees of freedom at the glenohumeral joint (78). Muscle activity at the shoulder is known to be highly coordinated and is largely driven by the need to delicately balance all joint torque requirements along with enforcing joint stability (12, 79). Due to the challenges with measuring and modelling shoulder kinematics (78, 80, 81), many upper extremity models may simplify representation of the shoulder by reducing the number of degrees of freedom. However, these simplifications must be informed by the research question. If the aim is to better understand neuromuscular control of the shoulder, reducing the kinematic degrees of freedom can eliminate many of the mechanical functions that muscles enforce. For instance, the rotator cuff muscles are important stabilizers of the glenohumeral joint (82). Removing glenohumeral translational degrees of freedom or not enforcing glenohumeral joint reaction force constraints can underestimate the activation of rotator cuff muscles as the stabilizing requirement for activating these muscles is not modelled (83, 84). Improving anatomic fidelity by adding kinematic degrees of freedom, such as the toes for lower limb models (85) or the wrist for hand models (86), will require secondary moments to be balanced and generally lead to higher levels of muscle co-activation (69). Similarly, choice of muscle pathway representation can influence the mechanical functions of a muscle and affect predictions of neuromuscular control strategies. MacIntosh & Keir (87) reported improved predictions of antagonist muscle activity when incorporating intrinsic finger muscles and extensor mechanism representation for an index finger model. Thus, conclusions on neuromuscular control using biomechanical models can be sensitive to model choices.

Individual differences in limb mechanics will additionally alter the feasible solution space that can cause inherent between-individual variation in muscle activity patterns required to perform identical tasks. Although few studies have explicitly investigated how the solution space may vary across individuals, substantial sensitivity in predicting musculotendon forces and muscle activations is reported with alterations in the muscle model parameters (88, 89), muscle attachment points (82, 90, 91), and kinematic definition of the model (42) [for a review on investigating stochasticity in model parameters, see (92)]. Further complicating matters, posture will non-linearly affect limb mechanics properties (e.g., lines of action, moment arms, force-length, pennation angle, inertia), resulting in alterations in the mapping from musculotendon forces to endpoint forces. For instance, cadaveric studies report changes in the fingertip endpoint force vectors generated when an individual tendon is loaded with the same magnitude across different postures (93–97). As a result, muscle coordination patterns required to perform the identical task are expected to vary within-subject as an individual changes their posture and between-subjects when comparing individuals who adopt different postures due to anthropometric differences.

Taken together, relationships in activation levels across muscles are shaped by the constraints of limb mechanics and task demands, creating inherent structure in the feasible solution space. The exact nature of these relationships is dependent on the individual's anthropometrics, anatomy, posture, limb, and task at hand. For most sub-maximal tasks, there is a large feasible solution space of possible muscle activity patterns that allow for the possibility of substantial between- and within-individual variation in motor behaviour.

## 5. Emergent muscle activity patterns

To this point, we have formally defined the degree of freedom problem and discussed theoretical solutions to the problem using the concept of the feasible solution space for muscle activations, which brings us to a key question: which solutions emerge when examining individuals in real life? That is, how do individuals inhabit and navigate the feasible solution space? Several studies have experimentally measured muscle activity patterns using EMG of the upper extremity during various static tasks. As expected due to changes in the feasible solution space, differences in EMG amplitude are observed with alterations in posture and task demands at the shoulder (98–102), elbow/forearm (103–107), and hand/wrist (17, 108–110). Variations in muscle activity patterns are also commonly noted within- and between-individuals. Interestingly, within-individual variability in EMG amplitude appears to increase with task intensity (111) despite the feasible solution space of muscle activations generally decreasing. This unintuitive finding is thought to arise due to the signal-dependent motor noise present within the nervous system (i.e., variability in EMG and force increases proportional to amplitude) (111, 112). The magnitude of between-individual variability is systematically larger than within-individuals (113), which is commonly hypothesized to represent individualized motor control strategies, partly due to differences in limb mechanics.

Upon further examination of the variability within- and between-individuals, despite the substantial magnitude of variability, muscles display relatively stereotypical coordination profiles as evidenced by extracting low-dimensional patterns (114–121). Although some structure in muscle coordination is to be expected due to mechanical constraints, the few studies making direct comparisons have found experimental data to exhibit more distinctive and less varying multivariate relationships across muscles than the set of all theoretically feasible solutions. During walking, the range of feasible activations for most lower limb muscles to balance net joint torques can range between natural limits (0%–100%). It should be noted that the feasible range of activations are likely to be reduced when considering the additional dynamical constraints that were not modelled (e.g., contraction-activation dynamics). Nevertheless, experimental muscle activity patterns display stereotypical patterns that fall within a relatively narrow band of activation levels (71). Similarly, experimental muscle activity patterns inhabited a small subspace of all feasible solutions during submaximal isometric finger pressing tasks (108). The small overlap in motor behaviour between what is observed experimentally in real-life vs. what is theoretically possible based on biomechanical modelling is incredibly meaningful and indicative of: (1) overarching neuromuscular control rules or principles governing the selection of certain muscle activity patterns and/or (2) additional neural and biomechanical constraints beyond just balancing net joint torques that are unaccounted for in current biomechanical models but may further shape muscle activity patterns.

## 6. Neuromuscular control theories: optimality, probabilistic control, and constraints

Several neuromuscular control theories (muscle synergies, uncontrolled manifold hypothesis, optimality, probabilistic control) are proposed to explain and predict voluntary control of muscles from the large set of possibilities, each of them elegantly encompassed within the framework of feasible solution spaces (2, 13). The muscle synergies theory proposes that the central nervous system organizes muscles into functional groups (i.e., synergies) that are tuned to certain task-level goals (117, 121). As a consequence, instead of coordinating every individual muscle, the central nervous system simplifies neuromuscular control by operating within a lower-dimensional space by recruiting muscle synergies to perform a wide range of tasks. An alternative perspective is the uncontrolled manifold (UCM) hypothesis, which suggests that the sensorimotor system can take advantage of the abundance of solutions by allowing elements (e.g., muscle activations and joint angles) to freely vary as long as errors in the performance variable(s) of interest, such as endpoint fingertip forces during pressing, are kept to a minimum (23, 24). In other words, the central nervous system is free to explore the available solutions inside the feasible solution space since they are all equivalent with respect to the mechanical demands of the task. Unlike muscle synergies, the UCM hypothesis embraces the high dimensionality of the musculoskeletal system. Interestingly, both muscle synergies and UCM hypotheses can emerge naturally by simply applying optimality and probabilistic principles to neuromuscular control (2). In this context, synergies are not a hard-wired constraint by the central nervous system (i.e., prescriptive synergies) but arise inherently due to the biomechanical constraints and optimization processes shaping the relationship across muscles (i.e., descriptive synergies) (13, 20, 22, 122). Furthermore, if the system's goal is to optimize task performance while minimizing effort, any deviations in degrees of freedom that have negligible effects on task-relevant variables can be ignored, resulting in an uncontrolled manifold along task-irrelevant dimensions where elements are free to vary (36, 37). The following sections will detail optimality principles and probabilistic control for promoting the preference of certain muscle coordination patterns and examine the evidence for neuromechanical constraints in limiting independent control of muscles.

### 6.1. Optimality

Optimality, as a neuromuscular control theory, proposes that sensorimotor behaviour is guided by overarching principles that aim to best meet neurophysiologically relevant criteria or goals. Fundamentally, it means that not every feasible solution is considered equal and that certain solutions are preferred. Optimality is an attractive idea, having parallels with many prevalent concepts across science (e.g., natural selection in biology, entropy maximization in chemistry). It would also be predictive of stereotypical patterns that fall within a narrow region of the feasible solution space, as consistent with experimental data of motor behaviour. In biomechanics and motor control, optimality principles have been the predominant approach over the past half-century for resolving the degrees of freedom problem, including the prediction of load-sharing across muscles and movement trajectories (123, 124). The two most common approaches are static optimization and optimal control. Static optimization is the partitioning of net joint moments, calculated using inverse dynamics, into individual muscle forces, and consequently muscle activations, independently at discrete timepoints (26, 27, 30, 52–54). Optimal control is a forward dynamics based method that finds the entire time history controls, typically muscle excitations, given an *a priori* cost function (consisting of 1 or more objectives) and a set of constraints (125, 126). Different optimal control methods exist [see (25) for a review of applications in neuromuscular control], with the majority of optimal control problems in biomechanics and motor control aimed at tracking or predicting movement (127–134). Constraints in these problems can be either incorporated indirectly through penalties in the cost function or directly enforced to be met. A less commonly used approach is inverse optimal control, which seeks to identify the cost function that best matches experimental data (typically movement trajectories) that is assumed to be optimal behaviour (31, 135). Optimal feedback control theory is a closed-loop application of optimal control that considers task goals and neurophysiological cost(s) while incorporating additive and/or multiplicative motor noise as well as delayed stochastic (i.e., noisy) sensory information with an internal feedforward model (35–37, 136). Integrating sensory information in a closed loop enables task performance despite model uncertainties and allows for online adaptations to instantaneous perturbations (e.g., motor/sensory noise, external environmental changes) (137) that can predict trial-to-trial motor variability as commonly observed in experimental data (37). The central challenge across these methods is identifying the one (or more) neurophysiological criteria that can be defined quantitatively as cost/objective function(s), which the system is presumably seeking to optimize for governing muscle and movement coordination. Although optimal control methods have successfully predicted many salient spatiotemporal features of movement trajectories using different cost functions, such as minimizing jerk (129, 138), torque (139), endpoint variance (140), and effort (37, 141), the focus here will be on predictions of muscle load-sharing.

Formulating a cost function to represent the sensorimotor system's goals has challenged researchers since early optimization models. Early work used linear optimization based on arbitrary, mathematically convenient objectives, such as minimizing muscle forces and stresses (force/cross-sectional area) (16, 52–54). The optimal solutions across these cost functions recruit muscles based on their moment arms and cross-sectional areas in a stepwise manner, with unrealistically large forces predicted in some muscles and minimal or no activity across several other muscles (28). The inherent limitations of linear optimization were also noted by Hardt (30), who found non-physiological results with the number of active muscles depending on the number of constraints modelled that are fundamentally unable to distribute forces across synergistic muscles. It was further suggested that physiological principles should be incorporated into the optimization process with the recommendation for a cost function based on muscle thermodynamics and minimizing the muscle energy requirements. Pedotti et al. (142) were able to predict forces distributed across muscles more reflective of experimental data using non-linear optimization (sum of square of muscle forces and normalized forces) but no physiological basis was provided for these cost functions. Combining the latter two approaches, Crowninshield & Brand (27) proposed minimizing the sum of muscle stresses raised to a power of 3 to reflect the sensorimotor system's possible goal of maximizing endurance during walking based on the non-linear inverse relationship between muscle stress and endurance. Similarly, Dul et al. (29) formulated a minimum fatigue criterion based on maximizing endurance accounting for muscle fiber type composition, reporting a better fit with experimental data compared to previously used cost functions. Since these initial modelling studies, non-linear effort-based cost functions (mainly quadratic and convex for ease of finding solutions) have remained the standard in biomechanics (123, 143). The definition of effort varies across studies, with most researchers electing for simple functions approximating what may be constituted as “effort”, such as muscle stresses, normalized muscle forces, or muscle activations (i.e., motor commands), while some studies include parameters underlying the metabolic processes of energy expenditure (127, 141, 144–146).

Coordinating muscles efficiently based on an effort criterion appears appealing and plausible. In addition to longer term evolutionary and developmental pressures (147, 148), several sources of evidence suggest that effort may drive motor behaviour. Direct evidence includes decreased muscle activity, co-activation, and metabolic expenditure (e.g., expired gas analysis) during learning of novel upper and lower extremity motor tasks (149–152). Indirectly, modelling simulations can successfully predict several spatiotemporal characteristics of reaching and locomotion kinematics using effort-based objectives (37, 127, 139, 141, 153). Studies making comparisons between the feasible solution space and EMG findings also report experimental muscle activity patterns to largely occupy the lower end of the feasible activation range for each muscle (71, 108). Although a causal link between effort and recruitment of muscles by the central nervous system is yet to be fully established, muscle coordination seems to be related (either directly or indirectly) with effort considerations. Nevertheless, the prediction of load-sharing across muscles by effort-based cost functions have proven controversial and unsatisfactory in many cases (123, 154, 155). Several studies observe a systematic failure to predict co-activation, even amongst the most advanced biomechanical models available today (156), particularly the under-prediction of antagonistic muscles by optimization-based models compared to experimental data. Based on the modelling framework presented, co-activation of muscles would simply increase the effort without altering the net joint moment, which would be “wasteful” mathematically using optimization. Caution should be made on whether the failure of optimization-based models to predict muscle co-activation may be due to choices made in model structure (16, 63–69) (see Section 4, paragraph 4). Nevertheless, the incompatibilities between effort-based optimization models and experimental observations for predicting muscle co-activation have been challenging to resolve but may be additionally explained by one or more of the following causes that will be highlighted in the subsequent sections:

a. **Globally Optimal vs. “Good-Enough”**: Is the central nervous system truly searching for the single best solution or are certain solutions deemed “good-enough”?
b. **Multiple Objectives**: Is the central nervous system simultaneously optimizing other objectives?
c. **Model Assumptions**: Are there additional neuromechanical constraints unaccounted for in the model?

### 6.2. Probabilistic control

While optimization may drive motor behaviour to explore more favourable solutions, the neuromuscular system may not identify the most favourable solution. The complexity of the high-dimensional feasible solution space limits the opportunity to exhaustively sample every solution and opens the possibility of considering “good-enough” solutions (39). “Good-enough” solutions generate costs that are comparable to the globally optimal solution, but not the best combination of costs that can be found (i.e., *approximately* optimal). A systematic exploration of the landscape of feasible solutions reveals that a diverse distribution of globally suboptimal muscle activity patterns can be found with similar costs (i.e., “functionally equivalent”) neighbouring the globally optimal solution in the cost function (i.e., fitness) landscape (34). Note that although different “good-enough” solutions may be “close” in the cost function space, they are not guaranteed to be “close” in the muscle activation space [see Figure 4b in (34)]. Convergence to different “good-enough” but functionally equivalent solutions may underlie individual variability in motor behaviour (34). The convergence to particular solutions may be determined by different initial states of the system (39), repeated exploration/sampling of the feasible solution space (157), motor variability (158), habitual behaviour (159, 160), and the diversity of adaptation strategies across people (161). Of interest, the exploration of the feasible solution space can be shaped by optimality principles. Greater variability in motor behaviour is commonly noted early on during motor learning, which may correspond with individuals exploring and searching for more rewarding solutions that are then exploited with decreasing variability over time (41, 152). As a result, individual-specific probabilistic representation of solutions are developed (i.e., multivariate probability distribution of muscle activities), characterizing the degree of belief (or uncertainty) in which motor solutions may work best for a given task (40, 41). Whether these solutions converge upon *the* optimal solution or one of several *approximately* optimal solutions, as well as how close to optimal constitutes “good-enough”, remain open questions (34, 39). Interestingly, the combination of “good-enough” solutions combined with strong prior probabilistic representations (40) may explain why muscle and movement coordination during some novel tasks remain similar to baseline motor tasks despite being suboptimal and individuals provided opportunities to practice the optimal solutions (see next section on multiple objectives as an alternative explanation) (159, 160, 162). In this vein, probabilistic methods can be a powerful approach for predicting muscle load-sharing that has yet to be fully exploited in biomechanics. Rather than finding the globally optimal solution with respect to effort, a range of possibilities can be predicted. These possibilities can be calculated using priors determined from the feasible solution space and adjusted based on the effort costs observed in the distribution of experimental muscle activity, allowing us to move past the shortcomings of simple optimization methods identifying only a single solution. For example, Markov Chain Monte Carlo methods with a prior weighed towards minimizing effort was recently used to find a distribution of plausible muscle activity patterns for a simple elbow flexion-extension motion (163). Probabilistic methods provide the benefit of capturing potential within- and between-individual variability in muscle activity patterns, uncertainty in motor control objectives, and identifying a range of possible outcomes when generalizing to novel experimental conditions (e.g., new motor tasks, neuromuscular impairments, rehabilitation, surgical outcomes).

### 6.3. Multi-objective optimization

The search for “good-enough” solutions and a probabilistic approach for predicting muscle load-sharing is especially promising in light of the growing evidence that the sensorimotor system optimizes multiple objectives. For example, optimal feedback control theory hypothesizes that sensorimotor behaviour balances the internal objective of minimizing effort with the external objective of maximizing task goals (i.e., reducing performance error) (36, 37). This can be evidenced by experimental studies observing greater muscle co-contraction during tasks requiring greater task accuracy (150). The central nervous system may also consider multiple external and internal objectives. One of the most well-known examples is the speed-accuracy trade-off that weighs two external task goals. More recently from the emerging field of neuroeconomics, decision-making during motor behaviour is found to weigh effort against rewards such that inefficient movements can be selected to elicit greater rewards (164, 165). Similar results are observed during decision-making weighing the relative costs of reaching and walking (i.e., preference for choosing paths requiring shorter reaching distances despite walking greater distances), which are found to be influenced by bottom-up biomechanical mechanisms (166, 167). Compared to any single measure of effort, multiple metrics of effort, including mechanical work and metabolic cost, were able to better estimate optimal strategy during split-belt adaptations (168). Using an inverse optimal control approach, a composite cost function combining minimizing effort and maximizing joint smoothness was able to better predict arm reaching trajectories compared to any single cost function alone (31). Likewise, a weighted cost function combining a number of objectives (cost of transport, muscle activity, head stability, foot-ground impact, and knee ligament use) resulted in closer agreement to healthy gait profiles (kinematics, kinetics, and electromyography) than any one objective (169). Although intuitively appealing, the implementation of multiple cost functions through a multi-objective optimization model is not trivial. Several cost functions may appear as appropriate neurophysiologically relevant objectives. A better fit between predicted and observed data may not necessarily reflect the explicit goals considered by the central nervous system and could be simple descriptors, but not necessarily causes, of motor behaviour (170). In fact, a multi-objective optimization model is expected to perform at least as well as single objectives since the weight of the additional objective can be zero if it adds no predictive value. Nevertheless, statistical comparisons between cost functions will allow us to start considering the likelihood of different objectives (170). An alternative approach to model-fitting with *a priori* cost functions is to design experimental paradigms that pit objectives against each other (171). Similarly, altering the cost landscape and identifying which solutions individuals converge towards during learning and adaptation may reveal how much weight (if any) the nervous system places on different objectives, which likely will vary across populations and tasks (158, 172, 173).

A plausible candidate for the consideration of additional objectives to the muscle load-sharing problem is mechanical impedance. The issue of redundancy has been at the heart of this review but is not the only “computational” challenge encountered by the sensorimotor system. Several additional problems inherent to the system are thought to affect sensorimotor control: noise, delays, uncertainty, nonstationarity, and nonlinearity (174). Stochastic sensory and signal-dependent motor noise (112) as well as uncertainties, either through imperfect sensory processing, incomplete environmental information, and unpredictable situations, will especially challenge our ability to perform tasks with accuracy. Interestingly, emerging evidence suggests that the majority of signal-dependent motor noise may not arise at the motor unit level due to stochastic motor unit discharge rates and unfused twitches as commonly thought, but rather an emergent feature of closed-loop feedback control (175–177). Nevertheless, control challenges related with stochastic noise and uncertainties can be compounded by delays in our sensorimotor system. Neural feedback pathways through short-latency (20–50 ms), long-latency (50–100 ms), and voluntary responses (>100 ms) may affect our ability to respond to mechanical disturbances in time (178). To combat these challenges, it is thought that the sensorimotor system uses feedforward mechanisms to maintain stability by actively controlling mechanical impedance, defined as a resistance to perturbations (45, 46, 179). Particularly important for postural control during isometric tasks is stiffness. Stiffness is the component of impedance accounting for resistance to changes in position and is quantified as the ratio between force and displacement changes (179–182). Impedance can be altered through changes in muscle activity that can affect the viscoelastic properties of individual muscles as well as the concurrent forces applied by several muscles (i.e., co-activation of synergists and antagonists). The increased joint stiffness with greater muscle co-activation outweighs the negative effects of signal-dependent motor noise resulting in an overall decrease in movement variability (150, 183, 184). In fact, co-activation of antagonist muscles can emerge as the minimal effort solution when incorporating motor noise and time delays using stochastic optimal control simulations (185, 186). On a related note, in a redundant task execution space, individuals find and exploit error-tolerant (i.e., robust) solutions, presumably so that variations in execution due to sensorimotor noise or perturbations will have minimal effects on task performance (57–59).

A couple studies to date have combined effort and mechanical impedance in a multi-objective optimization. Of particular relevance is a recent study investigating trade-offs between effort and limb stability in an isometric postural control task using a cat hindlimb model (34). The authors reported that effort and stability are competing objectives, resulting in a landscape of optimal solutions based on how much weight (0–1) is given to each term between globally minimizing effort and maximizing stability. The landscape of optimal solutions is termed the Pareto optimal set, where no solution can be found without making at least one of the objectives worse. Interestingly, it was found that small increases in effort from the minimal effort solution resulted in rapid increases in limb stability, indicating that low amounts of muscle co-activation can be an effective feedforward mechanism for increasing the mechanical impedance of a limb. The study did not collect any EMG, so no conclusions were made on the relative weight between effort and stability in real-life motor behaviour and whether these weights are generalizable across individuals and tasks. During an isometric elbow flexion task with fatigue, predicted muscle activations from a multi-objective optimization between minimizing effort and maximizing joint stiffness was strongly associated with collected EMG (*R*^2^ = 0.94) (33). The effort term was weighed more than joint stiffness but with fatigue, an increase weighting towards joint stiffness was observed and suggestive of alterations in the objectives of the central nervous system with changes in task demands. Joint stiffness has been a common focus in the spinal literature (187, 188) but has garnered lesser attention across other body parts. Intuitively, the shoulder would be a natural location for the central nervous system to weigh effort and stiffness together given the lack of passive restraints and reliance on active musculature for minimizing glenohumeral joint translations (12). Studies have implemented joint reaction force constraints at the glenohumeral joint based on cadaveric data for joint dislocation to confer stiffness (84, 189). However, these constraints still under-predict muscle co-activation, with the minimal effort solutions typically directed at the rim of the glenohumeral joint that could easily dislocate with perturbations or motor noise (83). Thus, an open question is whether neuromechanical factors, such as joint stiffness, are to be treated as objectives that are optimized vs. constraints to be kept at or within a certain level. There is some evidence to suggest that individuals exhibit greater levels of stiffness than the minimum required for stability during mechanically unstable force production tasks (46). Overlaying the neuromechanical costs (e.g., joint stiffness) that individuals exhibit during real-world tasks (190, 191) with the feasible solution space can help future studies tease apart the consideration of objectives vs. constraints.

In addition to task performance and effort considerations, muscles have a key mechanical function in injury prevention and progression that may be guiding neuromuscular control strategies. The active role of musculature in enforcing postural and limb stability does not only facilitate task performance, as discussed above, but also minimizes the risk of acute injuries, such as the prevention of falls and joint dislocations (192, 193). Of equal importance, muscles impose substantial internal joint-level loads that can mechanically contribute to the development of chronic musculoskeletal disorders (e.g., osteoarthritis) (194–196). For instance, hip reaction forces during walking are modelled to range anywhere from 1 to 10× body weight when calculating the feasible muscle activation space when only considering joint torques (74, 75). Given the role these mechanical loads play in chronic injury development, neural control of muscles may be tuned to not only maximize task performance (i.e., satisfy joint torque requirements of the task), but to also minimize internal joint stresses. Perhaps the strongest evidence to date indicating that neuromuscular control strategies may (in part) be regulated by joint-level mechanical loads is the recent work by Matthew Tresch and colleagues (197–199). Across a series of three experiments, quadriceps muscle activity patterns (vastus lateralis, vastus medialis, vastus intermedius, rectus femoris) were measured in rats to investigate their role in regulating mediolateral patellofemoral loads. First, across all locomotor task conditions tested (different slopes and speeds), the vastus medialis and lateralis exhibit the strongest pairwise correlations in muscle activity patterns (between all quadriceps muscles), likely reflecting a concerted neuromuscular effort to generate knee torque while balancing net mediolateral patella force (197). Furthermore, induced lateral loads on the patella through a spring promoted an adaptive decrease in the ratio of vastus lateralis-medialis activity (198). Finally, paralysis of the vastus lateralis resulted in a maintenance of vastus medialis activity and an increase in rectus femoris activity during locomotor tasks (199). In the latter study, if the rats compensated for the lost vastus lateralis torque by increasing vastus medialis activity alone, it would be at the expense of minimizing joint stresses (due to unbalanced net mediolateral forces). Alternatively, if vastus medialis activity decreased, substantial increases in rectus femoris activity would be needed to compensate for the lost knee extension torques from both the vastus medialis and lateralis; however, this could come at the expense of task performance due to the bi-articular nature of the rectus femoris that would require complex compensations at other joints. Thus, the experimentally observed maintenance of vastus medialis activity and moderate increase in rectus femoris activity in response to vastus lateralis paralysis is hypothesized to reflect a central nervous system goal of balancing the multiple objectives of minimizing internal joint stresses and maximizing external task performance (199). Others have similarly thought that the central nervous system regulates its neuromuscular control strategies to limit mechanical loads across joint-level tissues (17). Notably, muscle activity patterns that minimize joint loads do not necessarily align with optimization-based predictions of minimizing muscular effort, which has promoted the development of neurorehabilitation strategies to identify and train muscle activity patterns that minimize mechanical joint loads to limit the progression of pathologies (200–203). As a result, evaluating the delicate balance between the multiple competing neuromechanical objectives related to task performance, effort, and injury does not only offer a deeper understanding of human movement, but can also inspire novel strategies at injury prevention and restoring function in the presence of neuro-musculoskeletal pathologies.

### 6.4. Neuromechanical constraints: are muscles independent actuators?

In our modelling framework so far and as consistent with most biomechanical models, muscles are assumed to act as independent actuators. However, neural and mechanical factors can lead to force production and transmission between muscles (15, 204, 205). Perhaps the clearest example in humans is in the hand. Fingertip force production and/or movement of one finger leads to involuntary fingertip force production and movement of other digits (206–210). The relative contributions of neural vs. mechanical factors to the lack of muscle independence is often debated. Regardless, strong dependencies between muscle activations and force transmission would act as a constraint to the feasible solution space, such that certain combinations of muscle activations and forces are unattainable (e.g., maximal activation of index finger flexors without activating the middle finger flexors). Failure to account for neuromechanical constraints could lead to activity patterns substantially different than what may be predicted using optimization. Assumptions of independent control are thought to explain the poor prediction of finger forces during multi-finger tasks compared to individual finger tasks (143, 210). In the following subsections, we will review the evidence of mechanical and neural constraints limiting independent control of muscles.

#### 6.4.1. Mechanical factors

Mechanical constraints are attributed to passive tissue linkages between musculotendon structures [for a detailed recent review, see (211)]. The anatomical structures involved include connective tissue and neurovascular tract attachments between adjacent muscles and tendons, fascia surrounding muscle compartments, and intertendinous fascia (205, 211–213). As a result of these structures, force generation and length changes in one muscle can pull taut the connective tissue attachments, consequently leading to force transmission and altered force-generating capacity in neighbouring muscles.

Although the existence of inter-connections leading to interactions between musculotendon units is undoubted, their functional relevance remains uncertain. Early studies investigating musculotendon inter-connections in animal models observed differences in tendon forces between the origin and insertion sites, indicating force transmission outside the musculotendon unit. The tendon forces of a muscle can be further altered by changing the length and relative position of a neighbouring muscle (214–217). The functional contribution of these force transmission pathways during everyday behaviour is questioned (218), as manipulations to neighbouring muscles were beyond physiological ranges of motion. Follow-up experiments focused on manipulating limbs through the range of motion, particularly examining muscles crossing the knee and ankle. Within this experimental paradigm, knee flexion-extension angles were manipulated to produce length changes of biarticular muscles crossing both the knee and ankle (e.g., gastrocnemius). If forces are transmitted between the biarticular muscle and neighbouring uniarticular muscles crossing only the ankle (e.g., soleus), then different magnitudes of ankle moments across knee angles would be expected upon electrically stimulating the uniarticular ankle muscles. Yet, limited changes in ankle moments were found across knee angles among both cats and rats (219–221), with force transmission effects between muscles observed only when muscle groups were electrically stimulated at maximal levels (222, 223).

Conflicting evidence is also reported for the role of connective tissue linkages in limiting finger independence. Tensile forces applied to the tendon of one digit result in movement of adjacent digits (213). Removal of connective tissue linkages (e.g., intertendinous fascia, webbing) nearly eliminates movement at adjacent digits, supporting the mechanical function of intertendinous tissue in transmitting forces across fingers. Reinforcing these findings, passive motion of one digit at the metacarpophalangeal joint results in movement of other fingers (206), with similar magnitudes of involuntary movement observed during active motion (206) without any changes in motor unit activity (204, 207). In contrast, passive motion of the distal interphalangeal joint with anesthesia did not result in movement of other digits (204) and electrical stimulation of the extensor digitorum muscle compartments found relatively small magnitudes of force transmission to adjacent fingers (224).

Overall, the inconsistent evidence across studies suggests that assuming muscles are mechanically independent is non-trivial. Certainly, assuming that torque contributions from individual musculotendon units sum up linearly does not necessarily hold true. Several studies observe the phenomenon where electrically stimulating individual muscles and summing up their individual joint moment contributions does not equal the total joint moment when all muscles are simultaneously stimulated, indicating mechanical interactions between muscles (225–228). Furthermore, even if connective tissue attachments play a limited mechanical role in force transmission between muscles, spindle behaviour can be altered (229, 230), thereby affecting neural control of muscles through sensory pathways. Thus, a complex interplay between neural and mechanical factors, which are largely unaccounted for in current biomechanical models, emerges upon questioning whether muscles are independently controlled.

#### 6.4.2. Neural factors

Hard-wired neural pathways can constrain the ability to independently control muscles. Within the motor cortex, corticomotoneuronal cells projecting onto different muscles have overlapping regions (231–234). In addition, neural circuits in the spinal cord can diverge onto multiple muscles (235–237). Using viral retrograding techniques, premotor interneurons across the spinal cord are found to have monosynaptic connections with motoneuron pools of multiple synergistic and antagonistic muscles (236). As such, excitatory (or inhibitory) inputs can be delivered simultaneously to different motoneuron pools, which may underlie common synaptic input and neural drive to muscles (238). These circuits may form the neural substrates limiting independent muscle control but allow for muscle co-activation and co-inhibition to generate coordinated movement across limbs or regulate limb stiffness. In support of these neuroanatomical observations, synchronous discharge rates between pairs of motor units are observed within and between muscles (239–241), although whether common synaptic input is causing this synchronicity is questioned (242, 243). The differences in divergent synaptic connections across descending pathways may act as a further neural constraint to task performance. For example, the reticulospinal tract is highly divergent and is hypothesized to play a larger role in gross motor actions at the hand (e.g., grasping), as opposed to the less divergent corticospinal tract that can promote individuated finger control essential for fine motor tasks (e.g., manipulation) (244–247). The different functional roles of these descending pathways are speculated to compete against each other when met with the interacting mechanical demands of multiple tasks (e.g., postural maintenance vs. force control vs. movement) (47, 248, 249), which may require integration of separate neural control strategies (250, 251). The control strategies can be further complicated by the emergence of stochastic signal-dependent noise from the closed-loop aspect of motor control (175–177). Moreover, while control of joint torques is underdetermined (i.e., many muscle activity solutions for a given combination of joint torques), movement is driven by afferented muscles via spindles, rendering the control of muscle excursions as overdetermined (13, 51). That is, the length changes of many musculotendon units are determined by changes in fewer joint angles, leaving at best one solution for the control of muscle excursions. Amongst this backdrop of multiple constraints arising from the neural circuitry, the feasible solution space of muscle activity patterns will be reduced.

As an extension of neural constraints limiting independent control of muscles, some scientists hypothesize that the central nervous system recruits muscles in groups known as synergies (117, 121). A synergy is defined here as a functional unit comprised of muscles with weighted activations to represent muscles that are co-activated (or reciprocally inhibited). Each synergy is thought to be tuned to a specific task-level goal (107, 252, 253). Hence, instead of coordinating muscles individually, the central nervous system functions within a lower-dimensional space by recruiting muscle synergies to perform a wide range of tasks. The concept of muscle synergies parallels that of neural manifolds, where cortical activity during motor behaviour is observed to occupy a lower-dimensional space (i.e., manifold) (254, 255). Evidence for muscle synergies is mainly derived from stimulation of the brain and spinal cord (256–261). In these experiments, electrical stimulation of the central nervous system is observed to generate multi-joint torques that produce functionally relevant actions (e.g., reach or grasp). Supporting these findings, dimensional reduction statistics (e.g., principal component analysis, non-negative matrix factorization) reveal robust structural variations in muscle activity patterns that can largely be described by a small number of synergies (114–121). For example, Roh et al. (107) accounted for greater than 90% of the variation in EMG amplitude across 8 arm muscles during a submaximal isometric hand task across 210 force directions using 4 synergies. However, whether these synergies are “prescribed” by the central nervous system as a control scheme or are simply “descriptive” of the relationships between muscles is debated (13, 19–22). Based upon the same task conditions from Roh et al. (107), simulations performed using a musculoskeletal model with static optimization were able to re-produce synergies matching those found experimentally (262). This finding would be consistent with the argument that muscle synergies are not encoded as a control scheme but are a by-product of biomechanical constraints and learning optimal policies within the neuromuscular system (2, 20, 122, 263–265). Furthermore, although behaviour may be largely explained by a few groupings (i.e., synergies), the remaining unexplained variance that is often disregarded as noise may be under volitional control and required for task performance, manipulating compliant object interactions, or explaining control differences in clinical populations (266–270).

Regardless of the neural origin of muscle synergies, it appears that the neural circuitry provides at least some level of constraint on the voluntary control of muscles. The advantage offered by such constraints are to simplify the ability to control our high-dimensional neuromusculoskeletal system. Neuromechanical constraints may further serve to uniformly distribute stress across musculotendon units, reducing injury risk (271). In contrast, constraints to muscle control would decrease the feasible forces that can be produced at the endpoint of a limb and limit the ability to generate rich, flexible behaviour, such as adapting to novel environments, learning new tasks, or regulating limb stiffness (13, 72, 255, 272, 273). It is possible that the nervous system may be operating across both high- and low-dimensional spaces (274). For instance, the ability to independently control individual motor units varies from remarkable flexibility (275, 276) to rigidly constrained (277). Similarly, coherence analyses of motor units spike trains indicate that the motoneuron pool for a single muscle may not all receive the same common synaptic input, while motoneuron pools from different muscle compartments or groups may be constrained by the same common synaptic input (278–280). These observations have led to the recent viewpoint that muscles may not be the fundamental building blocks of neuromuscular control but rather “clusters” of motor neurons, striking a balance between flexibility and simplified control (281).

## 7. Conclusion

We started this paper by formulating the degrees of freedom problem using biomechanical modelling. The degrees of freedom problem is described as finding a solution within a large number of possible solutions, termed the feasible solution space. The feasible solution space is constructed based on modelled neuromechanical constraints, accounting for task demands and limb properties, and will include muscle activity patterns that are both optimal and suboptimal based on any theoretical cost function. Defining the feasible solution space does not mean that individuals are traversing across all the solutions when performing and learning a task. Rather, it identifies that individuals must be somewhere on this landscape. In fact, real-world motor behaviour, specifically muscle activity patterns in the context of this paper, appears to occupy only small regions of the feasible solution space. Perhaps the most fascinating question, one that scientists from many disciplines have been grappling with over the past century, is why individuals are choosing certain solutions within this wide landscape of sensorimotor possibilities. Underpinning this question are a conglomeration of interacting neuromechanical factors that may be shaping muscle activity patterns through the considerations of task performance, effort, and injury prevention. Although it may be a daunting task to piece together this apparent muddle of puzzle pieces, the challenges and opportunities available make the goal of better understanding neuromuscular control an exciting endeavour. As a scientific community, it is clear that our path ahead requires multi-disciplinary teams to meet at the intersection of biomechanics, neuroscience, and motor control. At the same time, we want to encourage readers to not let scientific progress be tracked solely by intellectually stimulating conversations within academic circles. Equally important is the ability to translate knowledge of neuromuscular control into the real-world, whether it is to inform neurorehabilitation strategies, develop safe workplace practices, or train athletes to maximize performance. After all, the ultimate measure of scientific success in our viewpoint is defined by the difference we make in human lives.
